# Hair Abnormalities in Genetic Disorders of Junctions

**DOI:** 10.4103/0974-7753.51926

**Published:** 2009

**Authors:** Paul D. Yesudian

**Affiliations:** Glan Clwyd Hospital, Denbigshire, UK

**Keywords:** Adherens, desmosomes, gap junctions, tight junctions

## Abstract

The desmosomes form the basis of intercellular support structure within the epidermis. However, various junctions, including gap junctions, adherens junctions, and tight junctions play an important part in the intercellular bridges that are vital for cell—cell interactions and structural stability. Numerous mutations can affect the genetic structure that make up these junctions and in turn cause disease. Most of these conditions have hair abnormalities and this article will briefly elucidate the various manifestations in the hair. As these junctional elements are found in other organs like the heart, liver, and eye, there could be serious systemic associations along with the hair changes.

## TIGHT JUNCTIONS

Tight junctions are made up of proteins called claudins, of which there are more than 20. They play an important role in paracellular communication and cell adhesion, and consequently in epidermal differentiation, proliferation, and barrier function.[1] Mutation of Claudin-1, first reported by Baala *et al*. in two inbred kindreds of Moroccan origin, results in diffuse ichthyosis, hypotrichosis, and scarring alopecia.[2] These patients also had sclerosing cholangitis and leucocyte vacuolization and the mutation was mapped to a 21.2cM interval of chromosome 3q27–q28. The comparison of mutant chromosomes in the two families suggested a common ancestral haplotype. Claudin 1 has also been identified in the liver and was recently recognized as a key factor for hepatitis C entry into the liver.[3] This emphasizes the importance of claudin 1 in the skin, hair, and liver biology.

## ADHERENS JUNCTIONS

These junctions differ from desmosomes in that they bind with actin filaments rather than intercellular filaments. The proteins in this junctions form a complex with α-, β-, and γ-catenins, which associate with the classic cadherins. A homozygous deletion mutation in the CDH3 gene (c981delG) that encodes P-cadherin causes hypotrichosis with juvenile macular dystrophy. It is an autosomal recessive disorder characterized by early hair loss which can be progressive. The hair loss is followed by severe retinal macular degenerative changes that culminate in blindness during the second to third decades of life.[4] P-cadherin is expressed in the hair follicle and in the retinal epithelium, contributing to this association. The genetic basis of the disorder has been localized to a gene defective in 16q22.1. This establishes the molecular basis of the condition and implicated a cadherin molecule in the pathogenesis of a human hair and retinal disorder.[5] Other homozygous mutations in the P-cadherin have been identified, but the constant feature in all these mutations is a clinical phenotype of hypotrichosis with juvenile macular dystrophy.

## GAP JUNCTIONS

A large number of connexin family proteins make up gap junctions, and they allow intercellular communications and the transfer of ions and other molecules between cells. They regulate a variety of physiological and developmental processes through this exchange of signaling molecules. Gap junctions are important in a number of different tissue types, with mutations causing diseases as variable as neuropathy, deafness, cataract formation, and skin disease. Mutations in connexin 30 have been reported to cause the Clouston syndrome (hidrotic ectodermal dysplasia), with the exact location mapped to a small interval on chromosome 13q11. This is a dominant form of ectodermal dysplasia and manifests as palmoplantar hyperkeratosis with alopecia and nail dystrophy. Patients have a diffuse hypotrichosis involving the scalp, eyebrows, eyelashes, and axillary and genital regions. It usually begins gradually, from childhood, although rarely it can be present from birth. Eye changes like cataracts and strabismus have also been associated with this syndrome, and some cases also have sensorineural hearing loss.[6] Other mutations in connexion 30 have been described that have shown features of keratitis, ichthyosis, and deafness (KID) syndrome with congenital atrichia.[7] Hence, the mutations of connexin 30 are associated with a spectrum of different phenotypic abnormalities.[1]

Mutations in connexin 43, coded for by the GJA1 gene, cause the oculodentodigital dysplasia. This is an autosomal dominant disorder with features of craniofacial (dental, nasal, and ocular) and limb dysmorphisms, spastic paraplegia, neurodegeneration along with syndactyly, brittle nails, and hypotrichosis.[8] Conductive deafness and cardiac abnormalities have also been observed rarely. A more recent heterozygous mutation in connexion 43, 780–781 del, has been described that shows curly hair with palmoplantar keratoderma.[9]

## DESMOSOMES

Desmosomal junctions are responsible for the adhesion of keratinocytes and are present throughout the epidermis increasing in size and number as they migrate up the epidermis. They anchor intermediate filaments at the membrane-associated plaques in adjoining cells thereby providing tissues with mechanical strength and maintaining cellular integrity. They consist of three families of proteins including the armadillo proteins (plakoglobulin and certain plakophilins), adherins (three desmocollins and four desmogleins), and plakins (desmoplakin, envoplakin, periplakin, plectin, bullous pemphigoid antigen 1, corneodesmosin, and microtubule actin cross-linking factor). They are also postulated to have a function in transducing signals. Mutations in the plakophilin 1 gene in p.Q304X and c.1132ins28 can result in skin fragility and inflammation.[10] The clinical features include erosions, fissures, and keratoderma with scanty hair and reduced sweating. This constellation was termed the ectodermal dysplasia-skin fragility syndrome. It demonstrates the importance of plakophilin 1 in the formation of desmosomal plaques.

Desmoplakin mutations were first described in an Ecuadorean family and manifest as plamoplantar keratoderma, dilated left ventricular cardiomyopathy and woolly hair (Carvajal syndrome). It is an autosomal recessive syndrome resulting from a truncated protein lacking the carboxy domain of its tail region (homozygous frameshift mutation, c.790delG).[11] Patients affected suffer from heart failure in their second decade of life. Clinicians must be aware of these associations due to this potentially serious outcome. Any patient with woolly hair and keratoderma must therefore be sent for cardiac evaluation.

Plakoglobulin mutations resulting from a homozygous two-base pair deletion, c.2157delTG cause a syndrome of arrhthmogenic right ventricular cardiomyopathy, non-epidermolytic palmoplantar keratoderma, and woolly hair. It is mapped to chromosome 17q21 and is inherited as an autosomal recessive condition.[12] The syndrome is prevalent in the Greek island of Naxos (therefore named Naxos disease). Plakoglobulin plays a role in signaling the formation of desmosomal junctions. These proteins are found not only in the skin and hair but also in cardiac myoctes. Mutations resulting in a truncated plakoglobulin therefore lead to detachment of the cardiac myocytes and ultimately myocyte death. Similarly, it causes desmosomal junctional fragility in hair shafts, explaining the woolly hair.

Desmoglein 4 mutations are mapped to the chromosome 3q with a deletion in EX5-8del, causing a localized autosomal recessive hypotrichosis.[13] Localized autosomal recessive hypothrichosis is a recently defined disorder characterized clinically with sparse hair in the scalp, chest, arms, and legs, but sparing of the axillary and pubic hair and eyelashes. Small itchy follicular papules can be seen on the scalp due to the inability of the hair to extrude through the skin. Desmoglein 4 is expressed in the hair follicle as well as suprabasal epidermis illustrating the dysfunction of epidermal and hair shaft integrity.

Corneodesmosin occurs within cornifying squamous epithelia and is expressed in the inner root sheath of the hair follicle. A heterozygous nonsense mutation in the gene CDSN (encoding corneodesmosin) causes an autosomal dominant hypotrichosis simplex.[14] In this condition, hair is normal at birth but progressive thinning of scalp hair begins within the first decade and is complete by the third decade. Other body sites are not affected. This hair loss is caused by aggregates of abnormal corneodesmosin accumulating around hair follicles and in the superficial dermis. The aggregates are toxic to hair follicle cells resulting in hypotrichosis simples of the scalp. This suggests that corneodesmosin is important in normal scalp hair physiology.

## CONCLUSION

A variety of hair disorders are associated with genetic disorders of the junctions. Understanding the microanatomy helps us to recognize the vital role they play in the formation of hair and other adnexal structures. Some of the syndromes have serious systemic manifestations and recognition of the genetic hair abnormalities is a clue to the clinician of the more potentially lethal nature of the disorder.
